# Discussing sexuality in the field of plastic and reconstructive surgery: a national survey of current practice in the Netherlands

**DOI:** 10.1007/s00238-018-1452-x

**Published:** 2018-08-18

**Authors:** Rieky E. Dikmans, Esmée M. Krouwel, Mahsa Ghasemi, Tim C. van de Grift, Mark-Bram Bouman, Marco J. P. F. Ritt, Henk W. Elzevier, Margriet G. Mullender

**Affiliations:** 1 Department of Plastic Reconstructive and Hand Surgery, VU University Medical Center, P.O. Box 7057, 1007 MB Amsterdam, Netherlands; 2 Department of Urology and Medical Decision Making, Leiden University Medical Center, Leiden, the Netherlands; 3 Department of Medical Psychology, VU University Medical Center, Amsterdam, Netherlands

**Keywords:** Plastic surgery, Sexuality, Quality of life

## Abstract

**Background:**

Patient-reported outcomes have become increasingly important to assess the value of surgical procedures. Sexual function is a proven important constituent of quality of life, but is often overlooked by health care professionals. We aim to investigate to what extent plastic surgeons address or discuss issues concerning sexuality with their patients, and if there is a need for improvement.

**Methods:**

We developed a survey to assess whether topics pertaining to sexual function were discussed during plastic surgical consultations. In 2016, all 385 members of the Dutch Association for Plastic Surgery were invited via post mail to participate.

**Results:**

We received 106 completed surveys (27.5%). The median age of the respondents was 45 (29–66) years. Most participants (78.3%) indicated that they rarely to never discuss sexuality with their patients. Surgeons in the subspecialization gender and genital surgery discussed sexual function most frequently. Two thirds of all respondents indicated that their current knowledge on this topic was insufficient, yet there was generally no interest expressed in receiving additional training (78.6%). However, there was a need for proper patient brochures (43.4%) and an organized referral network (36.5%) regarding sexuality.

**Conclusions:**

In plastic surgery practice, sexuality appears to be a rarely discussed subject, with the gender and genital surgery subspecialties as the exception. Although professionals and patients emphasize the importance of sexuality, plastic surgeons express limited urge to be trained and prefer written patient information and referring patients to other healthcare professionals. The authors stimulate more education on sexuality during (continued) plastic surgery training.

Level of Evidence: Not ratable

## Introduction

Health care is increasingly being assessed by the outcomes as experienced by patients. In recent decades, an increasing number of patient-reported outcome (PRO) measures have been developed to measure experienced outcomes [1, 2]. The primary overall outcome of many measures is the quality of life as reported by the patient. Quality of life comprises a number of constructs of which psychosocial well-being and physical health are well-known concepts. Sexuality is an important constituent of quality of life, but is often overlooked by health care professionals [3].

Diseases, medical treatments, and body image disturbances are all known to possibly negatively affect sexuality. Breast cancer patients, for instance, frequently experience sexual problems as a result of impaired body image [4]. The impact of (surgical) treatments on experienced measures of sexuality (e.g., sexual (dys) function, sexual activity, and satisfaction with sexuality) is only recently being explored and has been largely under-addressed by physicians [3]. The field of plastic surgery is dedicated to reconstruction of bodily defects due to birth disorders, trauma, burns, and disease. Many plastic surgeons perform cosmetic surgical procedures as well, which are focused on enhancing a patient’s appearance. Plastic and cosmetic surgery treatments typically have direct impact on esthetic appearance and may also affect sensation. Outcomes of plastic surgical treatments can be strongly associated with psychosocial factors including one’s body image [5]. Therefore, many plastic or cosmetic surgical treatments can also impact sexual function, which has been objectified for gynecomastia correction or cleft lip-palate surgery for example [6, 7]. In addition, it has been shown that the outcomes of breast reconstruction, which is the most frequently performed reconstructive procedure in Western society, are strongly related to measures of sexuality [4, 8].

Traditionally, (plastic) surgeons are primarily trained in the technical aspects of their profession. They are educated to deal with the physical problems, whether functional or cosmetic and their consequences for daily functioning. Addressing problems at another functional level, such as sexual function, requires additional knowledge, but also additional time. From former studies, we do know that addressing the topic is difficult for the patient as well as the physician due to several barriers including insecurity because of lack of knowledge [3, 9]. Presently, it is not known to what extent plastic surgeons address or discuss issues concerning sexuality with their patients. Here, we aim to identify the current plastic surgery practice in the Netherlands. In addition, we assess if there is a need for improvement from the plastic surgeon’s point of view.

## Methods

### Study design

In November 2016, a national survey was conducted in which all plastic surgeons and plastic surgery residents practicing in the Netherlands (*n* = 385) were approached via post mail to participate. The surveys were accompanied by an information letter and a post-paid return envelope. Addresses were obtained via the Dutch Society of Plastic Surgery (NVPC), which gave permission to send a one-off mailing only. Therefore, no reminders were sent. Data were collected and processed anonymously. Data collection was closed after 3 months.

### Development of the survey

The authors developed the survey in line with a previously developed instrument of similar kind [10]. The survey comprised 34 items, which focused on the background and experience of the plastic surgeon, as well as their practice related to discussing sexual functioning with their patients, their preferences with regard to sexuality training, and their interest in other sexuality support. The final survey included the following sections:

1. A demographic sheet assessing professional background (including interest areas within plastic surgery, clinical setting), years of experience in plastic surgical practice, gender, and age.
2. Several questions were asked about the frequency respondents discussed the subject of sexuality with their patients (at preoperative informed consent and postoperative follow-up consultations; 5-point Likert scale ranging from “never” to “always” and in percentages) and ways of discussing the subject (e.g., roles of team members).
3. A section on opinions about the importance of the topic of sexuality in their work (4-point Likert scales ranging from “not important” to “very important”), the responsibilities of the plastic surgeon, on past and ideal clinical training, and on (practical) barriers towards discussing the topic (“what is preventing you to talk about sexuality with your patients?”: e.g., patient age/ethnicity, duration of the consultation, insecurity or shame of the surgeon; disagree/neutral/agree answering options).

The present instrument was modified after a survey assessing similar subjects in another field of medicine [10]. A first version of the current measure, based on this scientifically valid tool described earlier, was tested in a pilot study in which five plastic surgeons provided feedback on the clarity and content of the questions. Based on their remarks, minor adjustments were made to the survey, resulting in the final instrument.

### Statistical analysis

Data analysis was performed using SPSS Statistics for Windows, Version 22.0. Armonk, NY: IBM Corp. Descriptive statistics were used to describe the outcomes. Equality of proportions between types of surgeons was tested with Pearson’s chi-square test or Mantel Haenszel test for trend, if groups were ordinal. Two-sided *p* values < 0.05 were considered statistically significant. In the questionnaire, surgeons could fill in more than one subspecialty. Per individual subspecialty calculations were made. Therefore, total sums of some analyses can add up to more than the total amount of participants.

### Ethical approval

As this study did not involve patients nor interventions and participation to this study was voluntarily, formal ethical approval was not required in the Netherlands.

## Results

### Participants

From a total of 385 members of the Dutch Society of Plastic Surgery, 106 plastic surgeons and residents returned a completed survey (27.5%). Two responding plastic surgeons stated they did not complete the survey because they considered the subject not applicable to their practice. The median age of the participants was 44 (range 29–66) years and 71.1% of the participants were male. The majority reported at least 5 years of experience in plastic surgery (91.5%); 14 respondents were residents in training (13.2%). Areas of interest and clinical settings are displayed in Table 1.

**Table 1:** Demographic characteristics (*n* = 106), *n* (%)

Age (range), median in years	44 (29–66)
Gender	
Male	76 (71.1)
Female	30 (28.3)
Experience (including residency)	
0–5 years	9 (9.5)
6–10 years	30 (28.3)
> 10 years	67 (63.2)
Function	
Plastic surgeon	92 (86.8)
Resident plastic surgery	14 (13.2)
Clinical setting	
University hospital	30 (28.3)
Top clinical teaching hospital	5 (4.7)
District general hospital	33 (31.1)
Private clinic	26 (24.5)
Categorical cancer hospital	1 (0.9)
Areas of interest*	
Breast reconstructive surgery (oncology)	77 (72.6)
Hand and wrist surgery	64 (60.4)
Cosmetic surgery	54 (50.9)
Head and neck reconstructive surgery	24 (22.6)
Genital surgery	19 (17.9)
Pediatric surgery	14 (13.2)
Burn reconstructive surgery	8 (7.5)
Gender surgery	5 (4.7)
Post bariatric surgery	2 (1.9)
Perianal reconstruction	1 (0.9)

*Multiple answers possible

### Discussing sexuality with patients

**Fig. 1:**
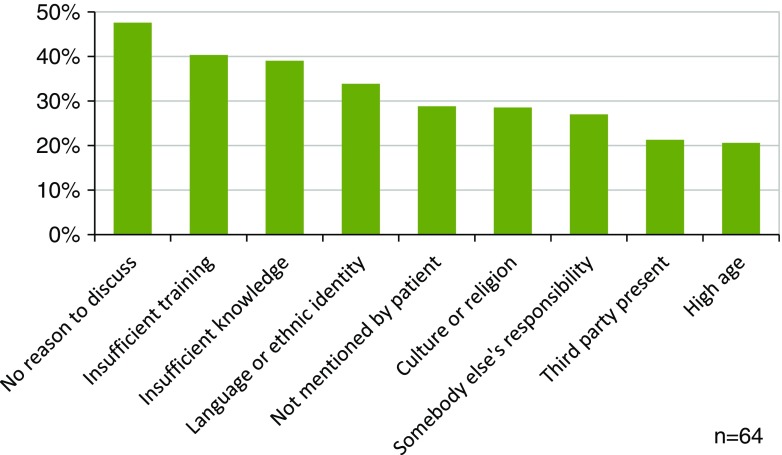
What prevents you from discussing sexuality with patients?

**Fig. 2:**
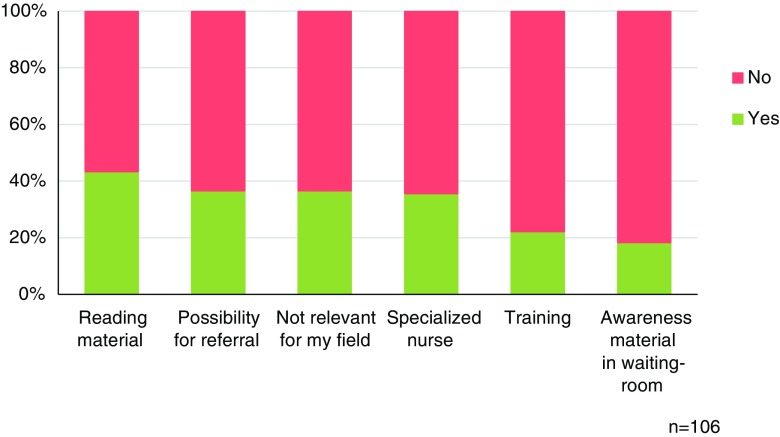
This could help me to discuss sexuality with patients

Most respondents (78.3%) reported they rarely or never discussed subjects regarding sexuality (Table 2). Both during preoperative informed consent consults as well as during clinical follow-up visits after surgery, sexual function was rarely or never being discussed (79.3%, 80.5%). When looking per subspecialty, plastic surgeons specializing in genital or gender surgery stated that they discussed sexuality with almost all patients. In all other subspecialties, this was the case in 5% or less of the patients (Table 3). When focusing on breast surgery specifically, cosmetic surgeons stated they rarely or never discussed sexuality with patients opting for breast reduction (55.2%) or breast augmentation (69.0%) respectively. In addition, 70.4% of surgeons rarely or never discussed the topic with patients who require breast reconstruction (Table 4). Yet, 61% of all responding participants mentioned that sexuality should be discussed at least once with patients undergoing breast surgery. More than half of the respondents (55.7%) stated that it is (very) important to inform patients about sexual complaints relating to surgical interventions. Twenty-six of the respondents mentioned they had referred at least one patient to a specialized sexuality care professional. When asked “what is preventing you to talk about sexuality with your patients?”, reasons that were confirmed most often were that there was no reason to discuss sexuality (47.6%), that they received insufficient training (40.3%), and that they experienced a lack of knowledge (40.3%) (Fig. 1). When being asked what could help the respondents to address sexual problems, “reading material for patients” was most frequently selected (Fig. 2). Among the respondents that did discuss sexual function, insecurity due to a changed self-image or appearance was the most frequently discussed topic (*n* = 41, 66.1%).

**Table 2:** Discussing sexuality with patients

	n*	(Almost) never	In less than 50%	In 50% or more	(Almost) always
How often do you discuss the patients’ sexual health?	106	78.3%	18.9%	0.9%	1.9%
Do you inform patients about consequences of surgery for sexual function during the informed consent procedure?	105	79.0%	16.2%	1.0%	3.8%
How often do you address sexual health during follow-up visits?	61	80.5%	12.2%	4.8%	2.4%
	n*	Not important	Somewhat important	Important	Very important
How important is it to inform patients about possible sexual complaints?	104	1.0%	43.3%	41.3%	14.4%

*Number of responders for this specific question

**Table 3:** In the past year, with which percentage of your patients did you discuss topics related to sexuality (per subspecialty)

Specialty	n*	Percentage Median (IQR)
Breast reconstruction	71	5 (15)
Head and neck	20	0 (0)
Gender	5	95 (25)
Genital	9	100 (0)
Hand and wrist	49	0 (0)
Burns	6	0 (6)
Cosmetic	47	5 (15)

*Number of plastic surgeons who treat patients within this subspecialty

**Table 4:** Discussing sexuality with breast surgery patients

How often do you inform women about (the consequences on) sexuality when they undergo					
	n*	Never	Rarely	Regularly	Often
- Breast reconstruction?	44	22.7%	47.7%	18.2%	11.4%
- Breast reduction?	29	41.4%	13.8%	27.6%	17.2%
- Breast augmentation?	29	34.5%	34.5%	17.2%	13.8%

*Only plastic surgeons working in the relevant subspecialty were included

### Responsibility, knowledge, and training

**Fig. 3:**
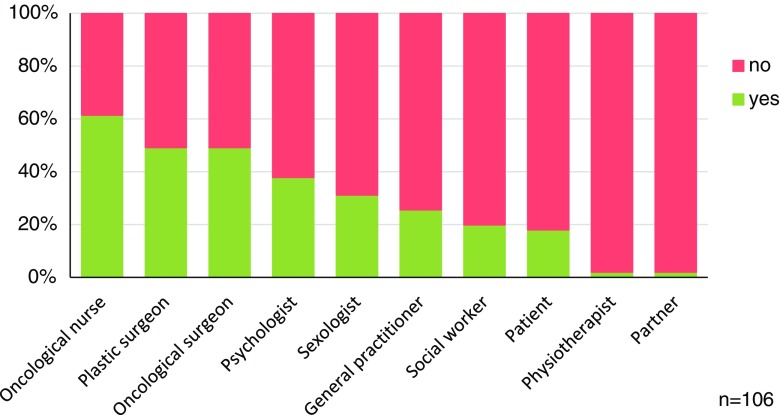
Who is responsible for raising sexuality as a discussion topic in the plastic reconstructive surgery practice?

Almost half of the respondents (49.1%) thought that plastic surgeons do have a responsibility to discuss sexuality-related issues with their patients. Although not applicable to all patient groups, oncological nurses and the oncological surgeon were also thought to have a responsibility to discuss the topic with the patient (Fig. 3). Only 6.1% of plastic surgeons stated that they had sufficient knowledge on sexual (dys)functions, while 86.2% stated that they had only little or no knowledge at all on the subject (Table 5). The majority of the respondents (64.7%) believed that sexuality was not adequately addressed during plastic surgery residency, yet only 6.1% underwent additional training. A minority of all participants (21.4%) was interested to learn more about the subject. This interest was significantly more expressed by participants who were still resident, when compared to plastic surgeons (50% vs. 16.9%, *p* = 0.01, Fisher’s exact test).

**Table 5:** Knowledge and training

	*n*	None (%)	A little (%)	Some (%)	Sufficient (%)
Do you have knowledge on sexual dysfunctions and treatments?	66	15.2	53.0	25.8	6.1
	*n*	Yes (%)	No (%)		
Do you think that sexology is adequately addressed during plastic surgery residency?	102	35.3	64.7		
Did you have additional training on how to address sexual problems of patients?	66	6.1	93.9		
Would you like to improve your skills with regard to addressing sexual health problems?	103	21.4	78.6		

## Discussion

The present study is the first to report on what role sexuality plays in the plastic surgeon’s consultation room. The data show that plastic surgeons infrequently discuss sexual functioning with their patients, with genital and gender subspecialists as the exception. Breast surgeons and cosmetic surgeons, two significant subspecialties within plastic surgery, generally agreed that sexuality is important for their surgery/population and that they carry a responsibility to discuss the topic. Still, many rarely discussed the subject with patients. Plastic surgeons experienced uncertainty on conversation starters, insufficient training, and limited knowledge as important barriers towards discussing the subject, and viewed the oncological nurse and psychologist as more appropriate team members to raise this topic. Hereafter, these findings will be discussed in the light of (1) the role of sexuality in plastic surgery practice, (2) how current practice on this topic relates to other specialties, (3) what structural barriers towards discussing sexuality in medical practice are currently known, and (4) how clinical services in plastic surgery may be improved regarding our present findings.

It is known that within the plastic reconstructive surgery population, sexuality can play an important role. Sexuality issues in general can derive from impaired body image, loss of sensation or (sexual) function of body parts, or partnership issues [5]. In breast cancer patients, for example, sexuality was found to be significantly impaired [4, 8]. This relationship between symptoms or consequences of surgery and sexuality also applies to other types of plastic surgery patient groups such as the people undergoing genital reconstructive surgery (incl. transgender individuals), cosmetic, burn, and even hand surgery populations [11–17]. Restoring an impaired (genital) body image can be a primary motivation for patients to opt for plastic reconstructive surgery [18–20]. In contrast to what patients may experience, many surgeons (possibly including many non-responders of this study) assume that sexuality is not an issue within their patient population.

Our data confirm that in current plastic reconstructive surgery practice in the Netherlands, sexuality is only rarely discussed. An explanation for this could be the existence of experienced boundaries to start the discussion, from both the patient’s and a surgeon’s point of view. Genital and gender surgeons indicated they integrate the topic more frequently than their colleagues from other relevant subspecialties such as breast surgeons. Possibly, this percentage was higher because of the surgeon’s assumption that sexuality is only relevant for surgeries in genital regions. However, the impact of other sexuality-related body parts should not be underestimated. Although sexuality applies to breast surgery very much [21], other medical specialties have also recognized the importance for sexuality in their practice, for example in urology, gynecology, but also in cardiology [10, 22–27]. Comparable studies to the present study in other fields of medicine show an equal lack of discussing sexology as well as the associated boundaries [10, 22–27]. It is positive that contemporary literature does emphasize these issues and attempts to invoke a responsibility among providers who treat patients with pathology in relevant areas. The discrepancy between patient experiences and physician assumptions underlines the importance of good basic knowledge in signaling of and counseling on sexuality issues within the plastic reconstructive surgery practice. It is important that surgeons are aware that sexuality can play a role within unexpected patient populations as well.

Findings in our study suggest that there exist structural barriers towards starting the conversation on sexuality within plastic surgery practice. These barriers may exist for both the patients and the health care providers. Earlier studies have found that the biggest barriers on this subject are formed by inadequate training, lack of knowledge, insecurity, and disbelieve in treatment options [28–30]. In other studies, it was shown that years of clinical experience, provider age, a history of training regarding sexual dysfunction, and an international setting of practice positively impact providers’ opinions and practices towards sexual issues of patients [23, 24, 27]. Also, fear of causing distress was found to be associated [25]. In our study, we confirmed many of the aforementioned factors for the Dutch plastic surgery practice. In addition, we also observed the existence of (false) assumptions regarding sexuality (e.g., “sex is not related to the condition that I treat,” “sexuality does not apply to certain age groups,” and “if the patients do not mention the topic, there is no issue”). In addition, the complexity of sexual function may not be sufficiently captured in the short time physicians have for their consultation [31].

Based on our findings, we can propose several suggestions to improve clinical services for future patients in plastic surgery with (possible) sexuality issues. We found that plastic surgeons and residents felt insufficiently trained on this topic and had little time to address the topic of sexuality with their patients. Also, respondents expressed a wish for written patient information material on this subject. In order to facilitate plastic surgeons in their discussion of this topic, it is essential to provide them with good patient information material that addresses the topic, lowers the threshold to discuss the topic, and provides all parties with good referral options [3]. In addition, we found that plastic surgeons feel that they carry a responsibility to signal and address sexuality. Subsequently, specialized psychologists or nurses best perform the treatment of existing sexuality issues. Oncology nurses for example have shown to play an important role in repeatedly question patients on this topic [9, 10]. Though, it is important to stress that this profession is not involved in the treatment of the non-oncological plastic surgery population. In these non-oncological patient groups, plastic surgeons do carry the responsibility to signal sexology issues. It is therefore helpful to collaborate interdisciplinary and provide a solid referral routing network. Plastic and reconstructive surgery is a multidisciplinary specialty and facilities already exist for non-sexuality domains. Judging from our results, we can expect more affinity with the topic from the younger generation of plastic reconstructive surgeons. Investing in (continued) training on sexuality and in the residency program can contribute as well. By initiating the discussion, clinicians have the potential to detect sexual dysfunction and to refer adequately when necessary, thereby improving overall quality of life of their patients [3, 26, 32]. Ideally, standardized outcome measures such as the BREAST-Q will further objectify this improved (sexual) quality of life [2].

The strength of this study includes the fact that it is the first nationwide survey on this subject and that we have reached a significant number of plastic surgeons from different fields. Limitations include the moderate response rate and number of missing data. The national plastic surgeons society permitted us to send only a single mail, which may partly explain the moderate response rate. Still, the response rate is comparable to other survey studies [10]. The included study population was relatively heterogeneous as no selection was performed based on subspecialty and/or years of experience (due to the study aim of generating an overview of the plastic surgical field as a whole). Therefore, plastic surgeons without interest in sexuality may not have responded, possibly making our findings less generalizable. In-depth interviews could help gaining a better understanding of the difficulties plastic surgeons encounter when they start talking about sexuality. For future studies, a larger number of participants could enable a more detailed analysis per subspecialty and/or other confounders such as years of experience, clinical training, and socio-cultural background. An example of such a study could be a pan-European study. At the end of the present survey, the proportion of missing data increased, most likely caused by the length of the survey and the detailed questions. Surgeons who do not integrate sexuality in their professional practice may have been less likely to complete the survey. Based on the present findings, a future survey should be shorter and cover the main topics only.

## Conclusions

In plastic surgery practice, sexuality appears to be a rarely discussed subject (with gender and genital surgery subspecialties as the exception). Although scholars and patients emphasize the importance of sexuality in postoperative quality of life, plastic surgeons express limited urge to be trained in this subject and prefer patient information and referrals. To improve early detection of sexual issues and create a safe space for patients to discuss the topic with their surgeons, the authors stimulate more education on sexuality during plastic surgery training.
